# EFFECTS OF DISCRIMINATION/VICTIMIZATION ON HEALTHCARE UTILIZATION VIA HEALTHCARE BARRIERS

**DOI:** 10.1093/geroni/igac059.168

**Published:** 2022-12-20

**Authors:** Krystal Kittle, Kyungmin Kim, Karen Fredriksen Goldsen, Kathrin Boerner

**Affiliations:** University of Nevada Las Vegas, Las Vegas, Nevada, United States; Seoul National University, Seoul, Seoul-t'ukpyolsi, Republic of Korea; University of Washington, Seattle, Washington, United States; University of Massachusetts Boston, Boston, Massachusetts, United States

## Abstract

Aging lesbian, gay, bisexual, and transgender (LGBT) adults are a health disparate population with unique healthcare challenges. Using data from the Aging with Pride: National Health, Aging, Sexuality/Gender Study (NHAS; N = 2,560), we examined contextual factors that influence the healthcare utilization of LGBT middle-aged and older adults. Causal indirect, direct, and total causal effects based on counterfactuals were computed to assess mediational links between discrimination/victimization and healthcare utilization via healthcare barriers. Discrimination/victimization had an indirect effect on health screenings via fear accessing health services both inside and outside of the LGBT community, financial barriers to care or medication, and being uninsured. Discrimination/victimization also had an effect on routine checkups and having a regular provider, via fear seeking health services outside of the LGBT community, financial barriers and being uninsured. Findings can be utilized in LGBT cultural competency trainings for health and human service providers serving aging LGBT people.

